# Hybrid simulation of pediatric gynecologic examination: a mix-methods study of learners’ attitudes and factors affecting learning

**DOI:** 10.1186/s12909-020-02076-7

**Published:** 2020-05-24

**Authors:** Anna Torres, Magdalena Horodeńska, Grzegorz Witkowski, Kamil Torres

**Affiliations:** 1 Department of Didactics and Medical Simulation, Medical University of Lublin, Chodźki 4, 20-094 Lublin, Poland; 2 Pediatric and Adolescent Gynecology Unit, University Children’s Hospital, Medical University of Lublin, Chodźki 4, 20-094 Lublin, Poland

**Keywords:** High Fidelity simulation training; teaching, Patient simulation, Gynecological examination, Paediatrics, Adolescent health, Hybrid simulation

## Abstract

**Background:**

Children and adolescents with reproductive health issues may require immediate or emergency care, however in many countries availability of the pediatric and adolescent gynecology (PAG) service is low**.** That being said, teaching PAG examination to OBGYN, pediatrics and emergency medicine residents seems reasonable, and cannot be underestimated. In order to provide residents with opportunity to learn PAG examination, a high-fidelity hybrid simulation workshop was implemented in our institution.

**Methods:**

The study aimed to investigate learners’ attitudes towards the high-fidelity simulation (HFS) hybrid model as compared with task trainer-SP (simulated patient)-voice model in the HFS environment and the factors connected to learners’ attitudes towards the hybrid model that could influence learning in high-fidelity simulation (HFS).

The concept of attitude was used as the theoretical framework and the mixed method approach to study design was utilized with simultaneous collection of quantitative (original questionnaires) and qualitative data (semi-structured interviews).

**Results:**

Residents valued the HFS hybrid model higher over task trainer-SP-voice model in regards to all three attitude components: cognitive (95%), affective (87.5%) and behavioral (83.7%). Analysis of qualitative data revealed six themes important to learners and informing learning of PAG examination in HFS. Further analysis of the themes allowed to develop a conceptual model, in which six factors connected to attitude components influenced learning. These factors were: task difficulty, attention, emotional realism of the simulation, patient’s emotions, physical realism of the simulation, and technical issues.

**Conclusions:**

Participants of our study appreciated learning experience with the HFS hybrid model more, based on attitude questionnaire. Moreover, findings revealed that multiple, various factors connected to attitude may influence learning of PAG examination in HFS with hybrid model, and we propose a conceptual model illustrating relationships between those factors.

## Background

Children and adolescents with reproductive health issues may require immediate or emergency care. However, many countries are deficient or lack the easy access to pediatric and adolescent gynecology (PAG) services. This may be in connection with the fact, that PAG education is structured into well-designed postgraduate programs in only few countries worldwide [1–3].

The PAG encounter differs in many ways from the adult one, therefore adult health care providers may not feel competent to conduct the PAG care [4]. It has been reported that anxiety and shame cause inhibition and reduce motivation to act, which may result in unnecessary referrals and delay of proper care [5]. There are many factors that make the PAG exam more complex and these include: additional procedural skills, high level of anxiety in a patient and her guardian, the need to adjust glossary used to explain the procedures, increased time and empathy devoted to procedure.

Therefore, teaching PAG examination to OBGYN, pediatrics and emergency medicine residents seems reasonable and cannot be underestimated.

Several teaching modalities described to train learners in adult gynecological examination skills, cannot be applied or are not as feasible in the field of PAG [6, 7]. To answer this need we have developed the high-fidelity simulation (HFS) hybrid model of PAG exam, which was described in details in our previous paper [8]. The present study aimed to investigate further this teaching modality by posing two research questions:

What were the learners’ attitudes towards the hybrid model as compared with pelvic trainer in the HFS environment?

What were the factors connected to learners’ attitudes towards the hybrid model that could influence learning in HFS?

The degree of immersion in simulation and emotional involvement may have an impact on the learning process. It is also proven that attitude influences behaviors and may have substantial impact on the learning-by-doing process, which is consistent with behaviorism and activity theory often used as theoretical bases of simulation-based education [9–13] Therefore the concept of attitude was used as the theoretical framework to investigated learners’ perception and factors that could influence learning with hybrid model of PAG. Using the mixed method approach to study design, we simultaneously collected quantitative and qualitative data enabling triangulation and complementation of data [14].

## Methods

### The setting, hybrid simulation-based model and participants

**Fig. 1:**
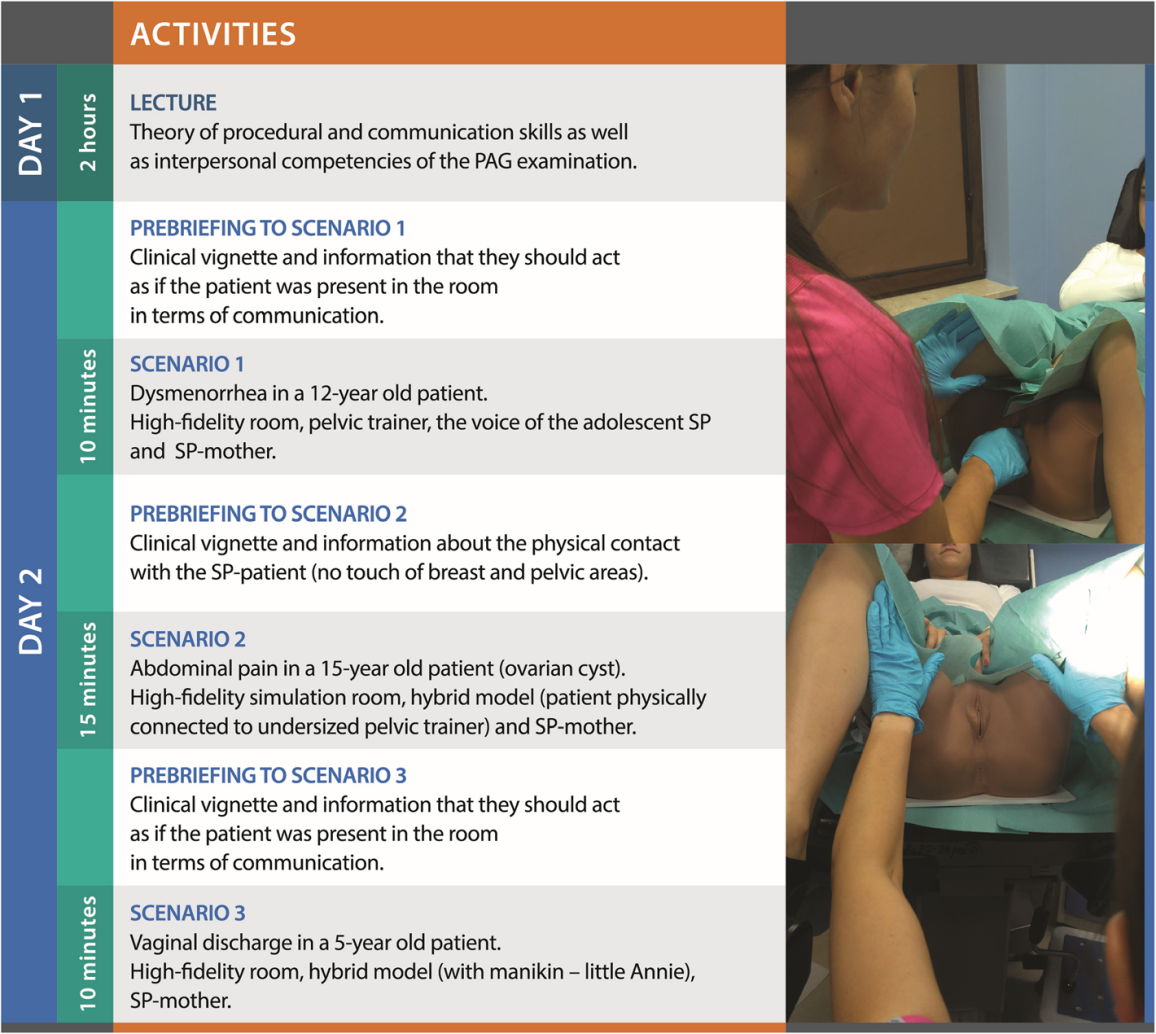
Outline of the teaching intervention (the PAG exam workshop) with the representative picture

The study was conducted in the Centre for Medical Simulation, Medical University of Lublin, Poland. The intervention was a 2-day workshop aiming to teach procedural, communication and interpersonal skills in PAG examination. The details of the intervention, the process of its development and SP training was described in details elsewhere [8]. The layout of the workshop and representative photo of the hybrid model was presented in the Fig. 1.

Sixteen residents agreed to take part in the study, of whom 11 were OBGYN trainees and five were pediatrics trainees. Majority of gynecology residents were in their first, second or third year of residency, and only five were senior residents. Pediatricians were in their second through fourth residency year. Thirteen participants were females. The mean age of the participants was 29.7 (SD 3.15; median 29) and varied between 25 and 38. Six residents had previous experience with simulation-based teaching, nine with pelvic trainers and none of them was taught with the participation of simulated patient (SP). In terms of their previous clinical experience in PAG: four participants performed at least one pelvic examination in an adolescent, and only one examined a prepubertal patient previously.

### Quantitative phase

The major part of the quantitative phase was devoted to evaluation of participants’ attitudes towards HFS hybrid model as compared to trainer-SP-voice simulation. However, we were also interested in self-perceived improvement in PAG skills as well as residents’ performance during scenarios with and without the HFS hybrid model.

The content of the original, web-based attitude questionnaire was based on the Tripartie Model, which defines attitude as composed of three components: affective, behavioral and cognitive components [9, 11]. Only verbal measures of all three attitude components: affective (5 sister-items), behavioral (7 sister-items), and cognitive (5 sister-items) were analyzed in the presented study (Supplementary file 1). Nonverbal measures of attitude, for example physiological responses of affect or recordings of overt behavior were not studied. By including sister items, we meant to minimize the possibility of subconscious suggesting and to check for quality and fidelity of answers. The items were rated on the four point-Likert scale including the following answers: strongly disagree-1, disagree-2, agree-3, and strongly agree = 4. The questionnaire is presented as a Supplementary file 1.

Self-perceived improvement in PAG skills was evaluated with the original, web-based questionnaire including 15 items rated on the 0 to 10 scale, which was completed twice: before and after the intervention (Supplementary file 2).

Direct observation of performance during scenario 1 and 2 was executed using the OSCE type checklist and global rating scale.

#### Statistical analysis

Using MedCalc software, medians median and interquartile range (IQR) were calculated, and differences were assessed using Wilcoxon signed-rank test for the paired comparisons and Mann-Whitney test for independent samples. The internal consistency of the questionnaires was calculated and expressed as Cronbach’s alpha. A *p*-value less than .05 was considered statistically significant. All tests were two-sided.

### Qualitative phase

Semi-structured interviews lasting 30 min each were conducted by one of the researchers (M.H.) with nine participants, who agreed to take part in this part of the study and were assisted by the interview guide, containing questions and probes on how the participants perceived the hybrid model in terms of a learning experience. The interviews were audio recorded and transcribed verbatim by research assistants. Data saturation was achieved after six interviews, however we decided to interview all participants; member checks were conducted. The data was analyzed independently by two of the authors (A.T. and M.H.) using thematic approach. Initial analysis of the data performed by each researcher yielded a coding framework of themes, which was used to code the transcripts in detail during a second round of analysis. In the iterative data analysis process, the coding framework was continuously adapted and discussed with a second researcher. A.T. and M.H. discussed their findings and found only minor differences. In a third analysis round the relations and meaning of the themes were analyzed and discussed in the research team and the conceptual model was developed.

## Results

**Fig. 2:**
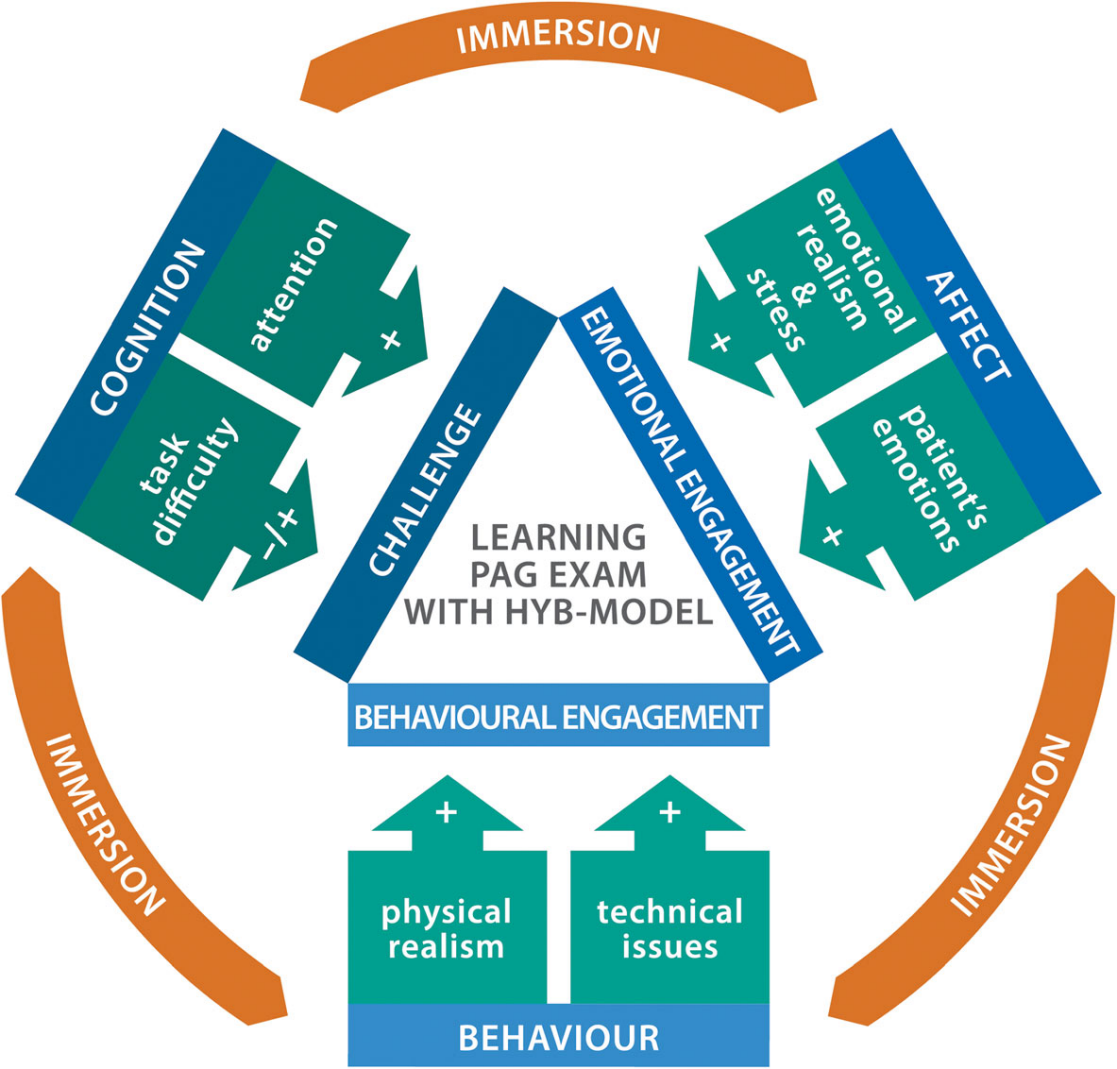
The conceptual model illustrating factors influencing learning in hybrid model high-fidelity simulation environment

Following our mixed methods approach, we present the integrated quantitative (Tables 1 and 2) and qualitative results for utilization of hybrid simulation in teaching PAG exam, with illustrative quotes from participants (Additional file 3) and conceptual model illustrating factors influencing learning in hybrid model high-fidelity simulation environment (Fig. 2).

**Table 1:** Results of the self-assessment questionnaire regarding skills in PAG examination. Each item of the questionnaire was scored on the 0 to 10 scale. The medians and the ranges for the self-assessment before and after intervention are presented as well as *p*-value for inferential statistics

Item No.	Item description	Median before (min-max)	Median after (min-max)	***p***
1.	Evaluation of development according to Tanner stage	5 (1–10)	6 (2–10)	0.034
2.	Assessment of adult female vulvar morphology	8 (0–10)	8 (4–10)	0.055
3.	Assessment of adolescent vulvar morphology	5 (2–9)	7 (4–10)	< 0.001
4.	Assessment of child vulvar morphology	2 (0–9)	5 (2–9)	0.002
5.	Assessment of clitoral morphology in a PAG patient	2 (0–8)	3 (2–9)	0.006
6.	Pelvic examination in an adult female	8 (0–10)	8 (0–10)	0.313
7.	Pelvic examination in an adolescent	2 (0–7)	7 (0–9)	0.001
8.	Pelvic exam of the girl below 12 years of age	0 (0–4)	5 (0–8)	< 0.001
9.	Sampling of vaginal secretion for microbiology testing in an adult female	8 (0–10)	9 (0–10)	0.094
10.	Sampling of vaginal secretion for microbiology testing in an adolescent	4 (0–10)	7 (4–10)	0.001
11.	Sampling of vaginal secretion for microbiology testing in a child	0 (0–7)	7 (4–9)	< 0.001
12.	Vaginal lavage for removing foreign body	0 (0–8)	5 (0–10)	< 0.001
13.	Communication with adolescent patient	5 (0–10)	8 (3–10)	< 0.001
14.	Communication with the child	4 (0–9)	7 (2–9)	< 0.001
15.	Global assessment of skills in PAG examination	5.5 (0–9)	7 (1–9)	0.019

**Table 2:** Results of the questionnaire inquiring about learners’ attitude towards hybrid model versus pelvic trainer-SP-voice model. The percentage of learners choosing each answer was presented as well as statistics for correlation test between answers to sister questions

Domain	Question No.	Strongly disagree %	Disagree %	Agree %	Strongly agree %	Correlation R (*p)*
Cognitive	5	0	18.8	50	31.2	**−0.802 (0.0003)**
	20	33.3	60	6.7	0	
	6	0	0	37.5	62.5	−0.382 (0.15)
	27	50	31.2	18.8	0	
	14	0	0	31.2	68.7	−0.416 (0.11)
	9	56.2	37.5	6.2	0	
	25	0	6.2	31.2	62.5	**−0.623 (0.01)**
	8	43.7	37.5	12.5	6.2	
	32	0	6.2	56.2	37.5	−**0.713 (0.002)**
	31	18.8	75	6.2	0	
Behavioral	2	6.2	0	12.5	81.2	**−0.627 (0.009)**
	12	56.2	37.5	0	6.2	
	4	0	12.5	43.7	43.7	−0.2 (0.46)
	17	12.5	31.2	43.7	12.5	
	7	0	0	37.5	62.5	**−0.637 (0.008)**
	18	37.5	56.2	0	6.2	
	13	6.2	25	37.5	31.2	**−0.648 (0.007)**
	23	37.5	50	12.5	0	
	19	0	31.2	31.2	37.5	**−0.543 (0.03)**
	10	31.2	50	12.5	6.2	
	22	0	0	33.3	66.7	**−0.567 (0.03)**
	29	31.2	62.5	6.2	0	
	24	0	6.2	43.7	50	−0.219 (0.42)
	16	18.8	37.5	31.2	12.5	
Affective	3	6.2	31.2	18.8	43.7	−0.406 (0.13)
	30	13.3	46.7	26.7	13.3	
	15	0	0	37.5	62.5	0.093 (0.73)
	1	56.2	18.8	12.5	12.5	
	21	0	0	50	50	**−0.728 (0.001)**
	11	43.7	43.7	12.5	0	
	28	0	12.5	37.5	50	−0.363 (0.17)
	26	56.2	37.5	6.2	0	
	34	0	12.5	56.2	31.2	**−0.594 (0.01)**
	33	12.5	75	12.5	0

### Participants’ attitudes: comparison between hybrid model and pelvic task trainer

During data evaluation the answers to questions pertaining to each of the three domains were combined and such analysis revealed that residents valued the hybrid model higher over task -trainer-SP-voice model in regards to all three attitude components: cognitive (95%), affective (87.5%) and behavioral (83.7%).

Analysis of individual items revealed that almost 63% of participants felt more anxious with the hybrid model as compared to trainer. In addition, 81% of participant felt it was easier for them to practice technical aspects of the exam with pelvic trainer. These observations suggest that hybrid model could more challenging due to stress comparable to the one present in real live conditions.

The Cronbach’s alfa for the questionnaire was 0.96. The correlation analysis of the items showed that coefficients for sister questions were negative for all but one pair of items and were statistically significant in 10 out of 17 pairs (Table 2). That indicated consistency in participants answers. The lack of statistical significance or inverse correlation was characteristic of the items pertaining to the affective domain and inquiring about participant’s anxiety and the difficulty of the task.

### Effects on acquisition of skills in PAG exam

Self-perceived skills in PAG exam increased significantly after the workshop, with no change noted in regards to an adult exam (Table 1). Significant differences in particular skills were noted between OBGYN and pediatrics residents. As it could be expected pediatricians scored themselves low in skills pertaining to adult examination and that did not change after the workshop. The differences regarding the PAG exam, which were present prior to intervention were leveled following the training (Table 1).

The Cronbach’s alfa for pre and post self-assessment questionnaires were 0.94 and 0.95, respectively.

The results of the direct observation assessment showed no difference between scenario-1 and scenario-2. Interestingly, no differences were observed between pediatrics and OBGYN residents either.

### Qualitative results

Analysis of the interviews uncovered six themes pertaining to factors that affect learning in the simulation environment using hybrid model of PAG examination. Illustrative quotes from participants pertaining to each theme were presented in paragraphs below and all quotes in the (Additional file 3).

#### Theme 1. The degree of physical realism and perceived task difficulty influenced learning

Participants perceived the hybrid model more real when compared with the pelvic trainer-SP-voice. The physical realism was perceived as factor that positively influenced learning and helped in developing complex skills.

*“I felt practicing with hybrid more real ... more serious...”*

*“...fidelity of simulation with hybrid model helps to perform the task...”*

At the same time the participant experienced that hybrid model made the task more challenging:

*“I think working with hybrid model was more challenging because of alive patient”.*

*“In my opinion practicing with hybrid is more challenging because you need to deal with two people”.*

Interestingly, the pelvic trainer-SP-voice scenario was not described as less challenging, but in terms of its realism participants perceived it as causing difficulty in learning of communication skills.

*“Communication was difficult to practice with trainer”.*

*“It felt weird to talk to plastic in case of the trainer scenario”.*

On the other hand, other skills like e.g. palpation of vulvar area were perceived as easier to execute using pelvic trainer-SP-voice model.

*“It is easier to perform exam without a human”.*

#### Theme 2. The degree of emotional realism influenced learning

It was often expressed that participants could experience SP’s emotions with hybrid model, which was not possible with pelvic trainer-SP-voice and that affected their learning.

*“...contact with living human can reinforce learning, especially reactions presenting discomfort...”*

*“It felt I gained more from the sim with hybrid, because of emotions, mimics and reactions of the simulated patient during the exam”.*

#### Theme 3. Participants’ emotional states during simulation were important for learning experience

Although the interview questions did not inquire about emotions, participants talked a lot about their feelings during simulations. The emotions experienced were stress and confusion. Stress was higher with hybrid model, and was perceived as positive or necessary element. Confusion was in part connected with the hybrid model and in part with the simulation as the learning modality in general.

*“I felt a lot of stress during the sim but I think this stress can be necessary ....”*

*“Definitely, I felt less stress with trainer”.*

*“I was not sure where does the trainer ends and SP begins – it made me confused”.*

Emotional involvement with the patient was also discussed. Participants said they felt emotionally involved only in case of hybrid model.

*“I must say I felt no emotional involvement with the patient in the scenario with trainer”.*

*“I was really emotionally involved with the case in the scenario where there was a hybrid model”.*

#### Theme 4. Comparison of the task difficulty between two types of simulation

This theme also emerged and was often outlined although participants were not asked about it directly and it was usually discussed in the connection to realism (mostly emotional).

*“It was much more difficult for me to perform task in case of hybrid model as it required combining various skills”.*

#### Theme 5. Engagement and attention with the patient were increased with hybrid model

Participants recalled deeper engagement and attention with the patient’s problems and experiences, when they performed PAG examination using hybrid model.

*“I felt more engaged in the scenario when the alive person was present in the chair; alive human is an asset”.*

*“I needed to pay more attention with hybrid model and felt more responsible for the patient”.*

#### Theme 6. Scenario with hybrid model was perceived as high-fidelity in contrast to the scenario with the trainer-SP-voice

Although both scenarios were conducted in high-fidelity environment with the only difference being the hybrid model, participants seemed to perceive “the fidelity” of the pelvic trainer-SP-voice scenario as low. They often used a term “trainer”, did not appreciate the SP voice coming from the speaker or even considered it a distractor.

*“The voice from the speakers distracted me during the sim with pelvic trainer”.*

*“With trainer it was possible to practice only technical skill - not communication”.*

## Discussion

Examination of the female intimate zone is difficult to teach in clinical environment especially in the field of pediatric and adolescent medicine. It was shown by several studies that skills difficult to learn with real patients could be successfully thought using simulation -based education, and that skills acquired through simulation were transferred to actual patient care situations [15]. High-fidelity simulation (HFS) has gained increasing interest despite large cost and effort, as it allows simultaneous mastering of technical, communication, and interpersonal skills combined with cognitive abilities allowing for timely and proper diagnosis and management [16]. The literature on simulation describes HFS environments as comprising of high-tech manikins, rooms resembling real clinical rooms, and finally the use of SPs, teaching assistants (non-doctor women instructors, professional SPs and trained women from the community, hybrid models and bystanders [16–21]. A review by Issenberg et al. points out, that HFS can be especially effective in regards to acquisition of complex skills [18]. Being a complex skill, the PAG exam undoubtedly belongs to those clinical situations that require teaching and learning in high-fidelity simulation environment. The recent study by Dumont revealed that PAG simulation training significantly increased residents’ knowledge and skills in major PAG competencies, and that it should be considered as part of the obstetrics and gynecology residency curricula [21].

The use of hybrid models linking a living human to a task trainer was described previously in various teaching contexts and proved its effectiveness in teaching complex skills in the field of obstetrics, urology, internal medicine, and procedural skills [19, 22–25]. Yet, to the best of our knowledge none of available studies has sought to identify how participants’ attitudes towards hybrid model could impact their learning. Our previous paper described development and incorporation of the PAG hybrid model to teach pelvic exam in pediatric patients and the overall residents’ satisfaction with that intervention [8]. The present study used a mixed-method approach and the Tripartie Model of attitude as the theoretical framework to determine, how the participant perceived the hybrid model in contrast to pelvic trainer-SP-voice model, when both were used in HFS environment, and how it influenced their learning [9, 11]. It has been suggested, that when learning gains are compared between simulation and “no intervention” the real role of the specific simulation methodology can be questioned and may fail to clarify the relationship between simulation fidelity and learning, and whether comparable gains might be achieved at substantially lower cost [26]. That is why in our study both models: the hybrid model and the trainer-SP-voice model were compared in the same group of participants and identical HFS environment. Analysis of the questionnaires results revealed that participants favored the hybrid model more as compared with pelvic trainer-SP-voice, and the difference was significant for all three attitude components: affective, behavioral and cognitive.

Qualitative data confirmed and further elucidated our quantitative findings. The themes extracted from participants statements helped us to understand, how both modalities influenced learning. Higher physical realism was an important factor connected with learning and influenced learning in a positive way, however, and not surprisingly participants perceived the hybrid scenario as more challenging. In addition, it was revealed, that learning specific task could be more or less problematic depending on the type of the model. For example, technical skills were easier to learn using pelvic trainer-SP-voice model, as it was perceived “less human”, whereas communication skills were more difficult to practice using this same modality. Our observations are supported by the study of Siebeck et al., who found that during HFS the training focused mainly on developing social skills required to perform the rectal examination, rather than on technical skills required to detect pathology [27].

Analysis of qualitative data uncovered that emotional realism, experiencing patient’s emotions and participants’ own feelings played important roles during learning in HFS, and differed between hybrid model and trainer-SP-voice model. This reveals an interesting relationship between emotional involvement and the engagement with case scenario, which seems to be based on bidirectional interaction between the SP (integrated with trainer in the hybrid) and the participant. This interaction apparently could not be achieved in trainer-SP-voice model. In order to prepare healthcare providers to real clinical situation HFS should have a high psychological fidelity and induce stress similar to real medical situations. Findings obtained during qualitative part of our study revealed, that stress was higher with hybrid model, however it was perceived as the positive and necessary component. None of the residents defined it as an element, which hindered learning. The work by Dias and Neto indicated that emergency medicine simulation may create a high psychological fidelity environment, similarly to what is observed in a real emergency room [28]. Seago et al. found that fear index before encounter with genital teaching assistant was high, however it reduced students’ anxiety and improved learner engagement with subsequent mechanical simulation practice of psychomotor skills [7]. The stress experienced by participants in our study did not seem to negatively influence learning as there were no significant differences in the scores assigned by the instructor during scenario 1 and 2. These findings support results presented by Siebeck et al., who argued that it might be possible to begin with complex learning tasks right from the start of the training curriculum, and at the same time seem to contradict the assumption that HFS may hinder learning, as it was claimed by Paas and van Merrienboer [29].

Interestingly, despite both scenarios were conducted in high-fidelity environment with the only difference being the hybrid model, participants seemed to perceive “the fidelity” of the pelvic trainer-SP-voice scenario as low. They often used a term “trainer” or “just plastic” referring to trainer-SP-voice scenario, and did not appreciate the voice of the SP coming from the speaker or even considered it a distractor. Although that observation was a surprise to us, especially that all participants had an opportunity to practice technical aspects of the exam before the scenarios in the task trainer laboratory, it is consistent with other authors’ observations, who argue, that terms like ‘integration’ or ‘presence’ may be more appropriate for describing closeness to reality than ‘fidelity’ [30, 31]. This is because fidelity is subjective relative to the task and the person performing it, which seems to be supported by the results of the presented study.

The validity of the findings reported in the article is limited to some extent by relatively small number of participants. However, we claim that they still can be of interest to the field of simulation-based education, taking to the account the PAG is a niche specialty. Additional limitation to data interpretation could be connected to the lack of information about the retention of skills as well as transferability to clinics. We plan our future studies to address those limitations.

## Conclusion

In conclusion, the study revealed, that participants of our study appreciated learning experience with hybrid model more, when compared to trainer-SP-voice model, although they were new both to PAG content and simulation environment. Moreover, findings from our study revealed, that factors connected to attitude influence learning of PAG examination in HFS with hybrid model, and we propose a conceptual model illustrating relationships between those factors.

We hope that findings of the presented study could contribute to better understanding of mechanisms influencing learning in HFS environments using hybrid models and begin wider dispute on the ways such models could be utilized in teaching and learning complex clinical skills. We also acknowledge that there are several areas that need further investigation regarding the use of hybrid simulation. These include transferability of skills to clinical setting, retention of knowledge, cost effectiveness and possibility of the use of minors as simulated patients.

## Supplementary information

**Additional file 1.** Attitude questionnaire.

**Additional file 2.** Self-assessment questionnaire regarding skills in PAG examination.

**Additional file 3.** Quotes from participants based on semi-structured interviews.

**Additional file 4.** The dataset supporting the conclusions of this article.

## Data Availability

The dataset supporting the conclusions of this article is included within the article and its additional files – Additional file 4.

## Abbreviations

PAG: Pediatric and Adolescent Gynecology
HFHS: high-fidelity hybrid simulation
HFS: high-fidelity simulation
OBGYN: Obstetrics and Gynecology
OSCE: Objective Structured Clinical Examination
