# Supplementary material for: Hybrid simulation of pediatric gynecologic examination: a mix-methods study of learners’ attitudes and factors affecting learning
Source: BMC Med Educ. 2020 May 24;20:164. doi: 10.1186/s12909-020-02076-7 (PMC7245870; doi:10.1186/s12909-020-02076-7)
Supplement: Supplementary file 1 — Additional file 1. Attitude questionnaire. [file 12909_2020_2076_MOESM1_ESM.docx]

**Participant No:________________________**

Age: _______________ Gender:

Residency _______________/ year:________

**Part A**

**Please answer the following questions:**

1. Have you ever taken part in training with participation of simulated patients ? Yes / No

2. Have you ever taken part in training with pelvic trainer? Yes / No

3. Have you heard about training with participation of simulated patients? Yes / No

4. Have you ever performed pelvic examination in an adolescent? Yes / No

5. Have you ever performed pelvic examination in a child? Yes / No

**Part B**

**Please state your position regarding following statements using 1 to 4 scale.**

| strongly disagree | disagree | agree | strongly agree |
| --- | --- | --- | --- |
| 1 | 2 | 3 | 4 |

1. When I practiced with trainer-SP-voice model it was easier for me to imagine I was dealing with a real patient situation
2. I prefer to practice PAG examination with hybrid model as compared to task trainer.
3. I was more stressed in the scenario with hybrid mode as compared to trainer-SP-voice model.
4. During scenario with hybrid model I behaved similarly as I usually do in a real patient encounter.

hybrydowego zachowywałem się podobnie jak zachowywałem się wcześniej wobec prawdziwych pacjentów.

1. I had more ideas about differential diagnosis when I practiced with the hybrid model.
2. When I practiced with the hybrid model I considered it a real patient.
3. It was easier for me to practice communication skills (verbal and non-verbal) with the hybrid model.
4. I think I learned more practicing with trainer-SP-voice model as compared to hybrid model.
5. I think pelvic trainer-SP-voice resembles real patient situation better that the hybrid model does.
6. It was easier for me to practice technical examination skills with the trainer-SP-voice model as compared to hybrid model.
7. My emotions towards trainer-SP-voice model were similar to those I had in a real patient situation.
8. I prefer to practice PAG examination with task trainer-SP-voice model as compared with hybrid model.
9. It was easier for me to initiate verbal communication with the patient’s mother when I practiced with the hybrid model.
10. I think that the hybrid mode is more realistic in resembling real patient encounter as compared to task trainer-SP-voice model.
11. When I practiced with the hybrid model it was easier for me to imagine I was dealing with a real patient situation.
12. When I practiced with the task trainer-SP-voice model I cared about the patient in the same way as I would care about a real patient.
13. During scenario with trainer-SP-voice model I behaved similarly as I usually do in a real patient encounter.
14. It was easier for me to practice communication skills (verbal and non-verbal) with the trainer-SP-voice model as compared to hybrid model.
15. It was easier for me to practice technical examination skills with the hybrid model as compared to trainer-SP-voice model
16. I had more ideas about differential diagnosis when I practiced with the trainer-SP-voice model.
17. My emotions towards hybrid model were similar to those I had in real patient situation.
18. It was easier for me to imagine how the patient felt during the exam when I practiced with the hybrid model
19. It was easier for me to initiate verbal communication with the patient’s mother, when I practiced with the trainer-SP-voice model
20. When I practiced with the hybrid model I cared about the patient in the same way I would care about a real patient.
21. I think I learned more through practicing with trainer-SP-voice model as compared to hybrid model.
22. I was more empathetic towards the trainer-SP-voice model than towards the hybrid model.
23. When I practiced with the trainer-SP-voice model I considered it a real patient
24. I was more empathetic towards the hybrid model than towards the trainer-SP-voice model.
25. It was easier for me to imagine how the patient felt during the exam when I practiced with the trainer-SP-voice model.
26. I felt more stressed, when I practiced with the trainer-SP-voice model.
27. I think, that training with the trainer-SP-voice model was more effective in preparing me for PAG exam in real patient situation, as compared to hybrid model.
28. I think, that training with the hybrid model was more effective in preparing me for PAG exam in real patient situation, as compared to trainer-SP-voice model.
29. Training with the trainer-SP-voice model made me more confident about PAG exam as compared to hybrid model
30. Training with the hybrid model made me more confident about PAG exam as compared to trainer-SP-voice model
