# Supplementary material for: Hybrid simulation of pediatric gynecologic examination: a mix-methods study of learners’ attitudes and factors affecting learning
Source: BMC Med Educ. 2020 May 24;20:164. doi: 10.1186/s12909-020-02076-7 (PMC7245870; doi:10.1186/s12909-020-02076-7)
Supplement: Supplementary file 2 — Additional file 2. Self-assessment questionnaire regarding skills in PAG examination. [file 12909_2020_2076_MOESM2_ESM.docx]

**Participant No:________________________**

Age: _______________ Gender:

Residency _______________/ year:________

**Please state your position regarding following statements using 0 (no skill) to 10 (proficient in skill) scale.**

1. Evaluation of development according to Tanner stage
2. Assessment of adult female vulvar morphology
3. Assessment of adolescent vulvar morphology
4. Assessment of child vulvar morphology
5. Assessment of clitoral morphology in a PAG patient
6. Pelvic examination in an adult female
7. Pelvic examination in an adolescent
8. Pelvic exam of the girl below 12 years of age
9. Sampling of vaginal secretion for microbiology testing in an adult female
10. Sampling of vaginal secretion for microbiology testing in an adolescent
11. Sampling of vaginal secretion for microbiology testing in a child
12. Vaginal lavage for removing foreign body
13. Communication with adolescent patient
14. Communication with the child
15. Global assessment of skills in PAG examination
