# Supplementary material for: Hybrid simulation of pediatric gynecologic examination: a mix-methods study of learners’ attitudes and factors affecting learning
Source: BMC Med Educ. 2020 May 24;20:164. doi: 10.1186/s12909-020-02076-7 (PMC7245870; doi:10.1186/s12909-020-02076-7)
Supplement: Supplementary file 3 — Additional file 3. Quotes from participants based on semi-structured interviews. [file 12909_2020_2076_MOESM3_ESM.docx]

Table 3. Quotes from participants based on semi-structured interviews.

| Theme | *Quotes from participants* |
| --- | --- |
| **Theme 1** | **The degree of physical realism and perceived task difficulty influenced learning** |
|  | *Q1: “It was easier to perform task in the scenario with hybrid model because everything seemed more real”*  *Q2: “One can practice everything with hybrid model”*  *Q3: “I felt practicing with hybrid more real ... more serious...”*  *Q4: “...fidelity of simulation with hybrid model helps to perform the task...”*  *Q5: “Interaction with patient is easier with hybrid model”*  *Q6: “I think working with hybrid model was more challenging because of alive patient”*  *Q7: “In my opinion practicing with hybrid is more challenging because you need to deal with two people”*  Q8: “For me gynecological exam was technically more difficult to perform due to patient reactions in scenario with hybrid”  *Q9: “...practicing communication in scenario with trainer was difficult – everything seemed less real”*  *Q10: “...with the pelvic trainer it was difficult for me to immerse in sim... too much to imagine”*  *Q11: “Communication was difficult to practice with trainer”*  *Q12: “It felt weird to talk to plastic in case of the trainer scenario”*  *Q13: “It is easier to perform exam without human”*  *Q14: “I think practicing communication with it (hybrid model) is highly appreciated - it reflects reality better”* |
| **Theme 2** | **The degree of emotional realism influenced learning** |
|  | Q15: “...contact with living human can reinforce learning, especially reactions presenting discomfort...”  Q16: “No visible reactions in scenario with trainer make it easier to concentrate more on technical aspects”  *Q17: “It felt I gained more from the sim with hybrid, because of emotions, mimics and reactions of the simulated patient during the exam”*  *Q18: “I think that patient’s reaction and emotions helped me learn and understand better”* |
| **Theme 3** | **Participants’ emotional states during simulation were important for learning experience** |
|  | Q19: “Definitely, I felt less stress with trainer”  *Q20: “There was a lot of stress with hybrid”*  Q21: “*I felt stressed because of being watched by other people – the patient mother and the instructor – I am not used to it”*  *Q22: “I was generally surprised and confused with simulation – it was first time experience for me”*  *Q23: “I was confused, what could be done with SP”*  *Q24: “I was not sure where does the trainer ends and SP begins – it made me confused”*  *Q25: “I must say I felt no emotional involvement with the patient in the scenario with trainer”*  *Q26: “I was really emotionally involved in the case were there was a hybrid model”*  *Q27: “I felt a lot of stress during the sim but I think this stress can be necessary ....”* |
| **Theme 4** | **Comparison of the task difficulty between two types of simulation.** |
|  | *Q28: “ ....communication with PAG patients and parents is difficult, I think it can be easier for pediatricians”*  *Q29: “It was much more difficult for me to perform task in case of hybrid model as it required combining various skills”*  *Q30: „With hybrid one needs to develop rapport with patient and mother, needs to communicate in a certain way and make the atmosphere to calm ... it is not easy”*  *Q31: “it was a real challenge to combine technical aspects and keep talking to both mother and a girl”* |
| **Theme 5** | **Engagement and attention with the patient were increased with hybrid model** |
|  | *Q32: “I think with Hybrid you pay attention to patient as a human being”*  *Q33: “ It was definitely easier for me to immerse in sim when the human was present – in hybrid model “*  *Q34: “I felt more engaged in the scenario when the alive person was present in the chair; alive human is an asset”*  *Q35: “I appreciate the possibility of performing full exam without invading patient’s privacy with hybrid model with interpersonal skills possible to learn along the technical skills”*  *Q36: „I needed to pay more attention with hybrid model and felt more responsible for the patient”*  *Q37: “ ...for me the presence of living human was important as it prompted more attention to patient and her behavior”* |
| **Theme 6** | **Scenario with hybrid model was perceived as high-fidelity in contrast to the scenario with the trainer-SP-voice** |
|  | Q38: “With trainer it was possible to practice only technical skill - not communication”  Q39: “The voice from microphone did not seem real”  Q40: “With trainer I felt it was just manikin, which did not help... adding at least a corpse or head would make it easier to practice communication, with the head you would know where to turn to, when you address the patient”  Q41: “The voice from the speakers distracted me during the sim with pelvic trainer” |
